# Tongguan capsule‐derived herb reduces susceptibility to atrial fibrillation by inhibiting left atrial fibrosis via modulating cardiac fibroblasts

**DOI:** 10.1111/jcmm.14022

**Published:** 2018-11-19

**Authors:** Shiyu Ma, Jin Ma, Liheng Guo, Junqi Bai, Shuai Mao, Minzhou Zhang

**Affiliations:** ^1^ Department of Critical‐care Medicine Guangdong Provincial Hospital of Chinese Medicine The Second Affiliated Hospital of Guangzhou University of Chinese Medicine Guangzhou China; ^2^ Guangzhou Key Laboratory of Myocardial Infarction in Chinese Medical Prevention and Treatment Guangzhou China; ^3^ Cardiac Electrophysiology Research Team Guangdong Provincial Hospital of Chinese Medicine The Second Affiliated Hospital of Guangzhou University of Chinese Medicine Guangzhou China; ^4^ New Patent Chinese Medicine and Decoction Pieces Innovative Research and Development Team The Second Affiliated Hospital of Guangzhou University of Chinese Medicine Guangdong Provincial Hospital of Chinese Medicine Guangzhou China

**Keywords:** atrial fibrillation, atrium, Chinese medicine, fibroblasts, fibrosis

## Abstract

Tongguan capsule is a compound Chinese medicine used to treat ischaemic heart diseases. This study aimed to investigate whether Tongguan capsule‐derived herb (TGD) has a preventive effect on atrial fibrillation (AF) in post‐myocardial infarction (MI) rats and to determine the underlying mechanisms. MI was induced by ligation of the left anterior descending coronary artery. TGD was administered to the post‐MI rats over a 4‐week period. The TGD‐treated rats had lower rates of AF inducibility and shorter AF durations than the MI rats. TGD improved the left atrial (LA) conduction velocity and homogeneity. It reduced the fibrosis‐positive areas and the protein levels of collagen types I and III in the left atrium. In vitro, it inhibited the expression of collagen types I and III by inhibiting the proliferation, migration, differentiation and cytokine secretion of cardiac fibroblasts (CFs). In conclusion, the current study demonstrated that TGD reduces susceptibility to AF and improves LA conduction function in rats with post‐MI by inhibiting left atrial fibrosis and modulating CFs. Targeting the CF population may be a novel antiarrhythmic therapeutic approach.

## 1 INTRODUCTION

Atrial fibrillation (AF) is the most common arrhythmia. It has become increasingly recognized as a major global health burden. Current therapies for AF include drugs and ablation, which treat symptoms and reduce the risk of tachycardia‐induced cardiomyopathy and stroke.^1^ Several large randomized trials have shown no benefit from antiarrhythmic drug‐derived rhythm control in terms of mortality. Existing antiarrhythmic drug approaches have limited effectiveness and are associated with the risk of serious complications, particularly proarrhythmia and organ toxicity,^2^ whereas ablation requires the destruction of viable tissue. Complications, costs and difficulties associated with ablation have encouraged the development of better and safer drug therapies.

Atrial fibrillation has a complex pathogenesis that is associated with underlying cardiovascular diseases, including myocardial infarction (MI) and heart failure (HF).^3^, ^4^ Increased fibrosis is observed in the extracellular matrix of the atrial myocardium during HF in both animal models^5^ and human biopsies.^6^ Fibrosis in the left atrium is a key substrate component for maintaining AF.^7^ Scientists have been investigating an upstream approach that can reduce atrial profibrotic signals, thereby preventing AF.^8^, ^9^ Accumulating evidence suggests that decreasing left atrial (LA) fibrosis in animal models can effectively inhibit AF susceptibility.^10^, ^11^, ^12^

A growing number of studies have demonstrated the protective cardiovascular effects of Chinese herbal medicines.^13^, ^14^, ^15^ Tongguan capsule (Chinese national patent number: CN101647856A) has long been used to treat ischaemic heart diseases, including acute coronary syndrome and angina pectoris in clinical practice.^16^, ^17^, ^18^, ^19^, ^20^ Previous data have shown that Tongguan capsules decrease infarct size, mitigate ventricular remodelling and prevent ischaemia/reperfusion arrhythmia occurrence in MI animal models.^18^, ^19^, ^21^ Tongguan capsule‐derived herb (TGD) consists of a Tongguan capsule formula (ie, *Astragalus mongholicus*,* Salvia miltiorrhiza*,* Hirudo medicinalis*) and adds *Ophiopogon japonicus*. Based on the results of ultra high‐performance liquid chromatography‐tandem mass spectrometry, TGD contains a variety of established cardioprotective ingredients, such as salvianolic acid B, salvianolic acid A and rosmarinic acid (Supporting Information), most of which demonstrate significant antifibrotic effects on different organ fibrosis models, including cardiac fibrosis.^22^, ^23^, ^24^ The present study aimed to investigate whether TGD can inhibit AF in post‐MI stage by reducing LA fibrosis and to explore the underlying mechanisms.

## 2 METHODS

### 2.1 Ethics statement

All animal procedures were conducted in accordance with the National Institutes of Health Guide for the Care and Use of Laboratory Animals and National Standard of the People's Republic of China for Laboratory animal‐Guideline for ethical review of animal welfare. During the experiment, the general conditions of animals were observed daily, including food or water consumption, weight, behaviour, infection and mortality.

### 2.2 The procedure of Tongguan capsule‐derived herb and quality control

Tongguan capsule‐derived herb consists of four traditional Chinese herbs, as shown in Table 1. All herbs were selectively chosen from genuine regional herbs obtained from Kangmei Pharmaceutical co. Ltd. (Guangdong, China), and identified through a thin layer chromatography (TLC) method (refer to Appendix VI B of Chinese Pharmacopoeia 2015 Edition). The reference standard herbs (*Astragalus membranaceus*: no. 121462; *Ophiopogon japonicus*: no. 121013; *Salvia miltiorrhiza*: no. 120923; *Hirudo medicinalis:* no. 121061) were purchased from the National Institutes for Food and Drug Control, China. In the chromatogram of all test samples, spots of the same colour were displayed on the positions corresponding to the chromatogram of the reference herbs.

**Table 1:** The information of Chinese medicines in Tongguan capsule‐derived herb

Chinese name	Latin name	Family	Place of origin (province)	Used part	Major effective compound in modern pharmacology study
*Huang Qi*	*Astragalus membranaceus*	*Leguminosae*	Gansu	Rhizome	Calycosin‐7‐O‐β‐D‐glucoside, Formononetin‐7‐O‐β‐D‐glucoside
*Mai Dong*	*Ophiopogon japonicus*	*Liliaceae*	Sichuan	Rhizome	Ophiopogonin D
*Dan Shen*	*Salvia miltiorrhiza*	*Labiatae*	Jiangsu	Rhizome	Salvianolic acid B, salvianolic acid A
*Shui Zhi*	*Hirudo nipponica Whitman*	*Hirudinidae*	Shandong	Dry whole	

Chinese medicines’ dosage ratio is 3:3:2:0.3 (*Huang Qi: Mai Dong*:* Dan Shen: Shui Zhi*).

Briefly, after drying, these herbs were mixed in proportion and macerated overnight at room temperature with distilled water 10 times (v/w). Then, the entire mixture was decocted twice for 2 hours each time. The filtrates were mixed and condensed at −0.09 Mpa, 60°C to a final concentration of 1 g/mL (weight of the original herbs/vole of solution). The filtrate solution was slowly added to 95% ethanol to a final concentration of 70% ethanol, precipitated for 24 hours under refrigeration (4°C to 8°C), and then dried through spray drying at 60°C. The mean yield of TGD was 17.2% (w/w), according to the original herbs. The resulting powder was stored in sealed containers at −20°C and diluted to the concentrations needed with distilled water and filtered before use. The quality control of the TGD and compounds identification is described in the Supporting Information.

### 2.3 Animal model and drug administration

The MI model was established using the method previously described.^25^ Male Sprague‐Dawley rats (weight 250‐280 g, SPF, Guangdong Medical Experimental Animal Center) were anaesthetized. Intubation and mechanical ventilation were subsequently performed with a small‐rodent ventilator (Harvard Apparatus, Holliston, MA, USA). With the heart exposed by a 1.2 cm lateral thoracotomy, the left coronary artery was permanently ligated 2 mm below the tip of the left auricle with 6‐0 nylon silk. The MI model was considered to be successfully established when myocardial blanching and cyanosis were visualized within the downstream myocardium. Thorax closure was performed with three layers of sutures. The sham‐operated animals (sham, n* *=* *15) underwent the same procedure except that the silk suture was placed around the left coronary artery without being tied. The mortality rate during and 7 days after the MI surgery was 30%. No deaths were observed in the sham animals. In the infarcted groups, 20% of the animals died from intraoperative arrhythmia during the surgery, and 10% of the animals died during the recovery period. One week after the MI surgery, only the survived rats with LV ejection fraction (EF) <45% performed further experiments. The rats were randomly grouped into MI group (MI, n* *=* *15), low‐dose TGD group (TGD‐L, n* *=* *15) and high‐dose TGD group (TGD‐H, n* *=* *15). There was no significant difference in cardiac function between the three groups (Supporting Information). TGD rats were gavaged at a dose of 0.6 g/kg/d in the TGD‐L group or 1.2 g/kg/d in the TGD‐H group for 4 weeks, which was derived from the equivalent conversion between animals and human by body surface area, based on the recommended daily human dosage of Tongguan capsules and according to a previous study of Tongguan capsules in rats.^26^ Other rats were gavaged with an equivalent volume of water. No deaths were observed after 4 weeks of treatment in any of the groups.

### 2.4 Programmed electrical stimulation technique

After 4 weeks of TGD or water treatment, induction of AF measurement was performed, as we described previously.^27^ AF was defined as irregular, rapid atrial activation with varying electrogram morphology lasting ≥2 seconds. The atrial rates were typically >1500 bpm in rats, as described previously.^28^, ^29^ The trachea was intubated via the mouth and mechanically ventilated with a small‐rodent ventilator (Harvard Apparatus, Holliston, MA, USA). For atrial stimulation, a 4‐French quadripolar catheter was advanced through the esophagus and placed at the site with the lowest threshold for atrial capture. Atrial pacing was performed at twice the diastolic threshold using two electrodes on the pacing catheter. Inducibility of atrial arrhythmias was tested by applying 35‐second bursts, using the automated stimulator. The burst had a cycle length of 20 milliseconds and pulse width of 5 milliseconds. This series of bursts was repeated once. All rats were allowed 5 minutes of recovery in the sinus rhythm between stimulations for respiratory and circulatory recovery. AF was considered inducible if one or more bursts induced an episode of AF. Otherwise, AF was considered to be non‐inducible. The longest record time was 30 minutes after the burst pacing. Data were sampled using Labchart software (AD Instruments, Sydney, Australia). Heart rate, RR interval, the duration and probability of inducible AF episodes were analysed using Labchart software (AD Instruments). Mean AF duration was based on the duration of rats with inducible AF.

### 2.5 Multielectrode array measurement

After the induction of AF measurement, multielectrode array (MEA) measurement was performed as we described previously.^27^ The heart was removed rapidly, and the left atrium from the isolated heart was separated and then immersed in Tyrode's solution. For MEA mapping, the epicardial LA surface rested on the MEA (Multi Channel Systems, Reutlingen, Germany) culture dish containing 120 tipped platinum recording electrodes of diameter 30 μm with an interelectrode spacing of 100 μm, and continuously superfused at a flow rate of 3 ml/min with oxygenated modified Tyrode solution with at 37°C.^30^ During recordings, contractility was blocked with 15 mmol/L butanedione monoxime (BDM). The electrode arrays were mounted onto a printed circuit board and then fitted into the MEA 2100 System interface (Multi Channel Systems). Data were sampled at 10 kHz per channel with simultaneous data acquisition using an MEA 2100 System (Multi Channel Systems). Electrical stimulation (bipolar pulses, 1‐7 V, 1000‐μs duration, 5 Hz frequency) was applied via one of the MEA microelectrodes. Data were sampled at 10 kHz per channel with simultaneous data acquisition using the Cardio 2D software (Multi Channel Systems), and five fields were recorded in each atrium. All the data were analysed to generate activation maps and measure conduction velocity (CV) using the Cardio 2D+ software (Multi Channel Systems).

### 2.6 Histology

Masson's trichrome staining of the Paraffin section prepared from bouin's fixed samples was performed as previously described.^31^ After the induction of AF measurement, the heart was arrested in diastole by injecting 1.0 mL of 10% potassium chloride intraperitoneally and fixed in 4% paraformaldehyde for 24 hours. Cryosections (5 μm) were stained with Masson trichrome. Images were digitized using a digital camera (DP 72; Olympus, Tokyo, Japan) under a BX53 microscope (Olympus). Images were quantified by the CellSens Dimension 1.16 software (Olympus). The fibrosis area was expressed as a percentage of red‐positive stained area to the total tissue area using the software.

### 2.7 Cell culture

Cardiac fibroblasts (CFs) were obtained from the adult rats’ atrium using the enzymatic dissociation and characterized as previously described.^31^ After rinsing in cold phosphate‐buffered saline (PBS), the heart was minced and digested with trypsin and collagenase type II (Gibco BRL Co. Ltd., Grand Island, NY, USA) at 37°C. After a 1.5 hours period of attachment to uncoated culture plates, the cells that were weakly attached or unattached were discarded, and the attached cells (mainly fibroblasts) were washed and grown in Dulbecco minimum essential medium (DMEM) supplemented with 10% foetal bovine serum (FBS). The TGD could dissolve in water. We used the 4 and 5 mg/mL TGD for the cellular experiment.

### 2.8 Proliferation assay

Cell proliferation was assessed by the 3‐(4, 5‐dimethylthiazol‐2‐yl)‐2, 5‐diphenyltetrazolium bromide (MTT) assay. The assay was based on the transformation of the tetrazolium salt MTT by active mitochondria into an insoluble formazan salt. MTT was added to each well under sterile conditions, and the plates were incubated for 4 hours at 37°C. Untransformed MTT was removed by aspiration, and formazan crystals were dissolved in dimethyl sulfoxide (100 μL/well). Formazan was quantified at 570 nm using an Epoch multi‐volume spectrophotometer system (BioTek, Winooski, VT, USA).

### 2.9 Cell cycle analysis

To determine the effect of TGD on the cell cycle, CFs were seeded and incubated at 37°C overnight. Then, the cells were exposed to indicate concentration of TGD. Cells were collected and fixed with ice‐cold 70% ethanol and kept at 4°C overnight. Thereafter, the cells were collected and washed with PBS. The cell pellets were resuspended and stained in PBS containing RNase A and PI for 30 minutes at room temperature. Cell distribution across the cell cycle was determined with a BD Accuri C6 flow cytometer (BD Biosciences, San Jose, CA, USA).

### 2.10 Cell apoptosis assay

Apoptosis was analysed by flow cytometry analyses using fluorescein isothiocyanate (FITC) Annexin V Apoptosis with propidium iodide (PI) Detection Kit (BD Biosciences). After receiving different treatments for 48 hours, CFs cells were harvested, washed and resuspended in a binding buffer. Then, the cells were incubated with Annexin V‐FITC and PI in the dark for 15 minutes. Finally, binding buffer was added, and the stained cells were analysed using a BD Accuri C6 flow cytometer (BD Biosciences). Q4 represents normal cells, and the early and late apoptotic cells are located in the Q3 and Q2 regions. The necrotic cells are distributed in the Q1 region. The relative ratio of early and late apoptotic cells was chosen for further comparison.

### 2.11 Migration assay

Cell migration capacity was determined using a Transwell chamber (8 μm pore size; Corning, Corning, NY, USA), according to the manufacturer's instructions. Briefly, a suitable number of CFs were placed on each upper chamber in 200 μL of serum‐free DMEM. In the lower chamber, 500 μL of complete medium (containing 10% FBS) was added with TGD. After 24 hours of incubation at 37°C in 5% CO_2_, cells in the upper surface of the membranes were removed. The cells that had migrated through the pores and attached on the underside of the membrane were stained with Leucocrystal Violet. At least six randomly selected images were counted, and the average number of stained cells represented the relative migration.

### 2.12 Immunocytochemistry assay

Cardiac fibroblasts were plated on cover slips in 3‐mm culture plates, treated with TGD for 48 hours, fixed with 4% paraformaldehyde and permeabilized with 0.3% Triton X‐100. They were blocked with 10% normal goat serum and incubated with primary antibodies to vimentin (1:200; Biotechnology Inc., Santa Cruz, CA, USA) and α‐smooth muscle actin (α‐SMA, 1:50; Abcam, Cambridge, UK). After incubation with the primary antibodies, the cultures were rinsed with PBS and then incubated with fluorescence‐labelled secondary antibodies. Nuclei were stained with 4, 6‐diamidino‐2‐phenylindole (DAPI).

### 2.13 Hydroxyproline assay

The total collagen content in equal amounts of culture supernatants was analysed with a hydroxyproline assay using a commercially available colorimetric assay kit (BioVision Inc., Milpitas, CA, USA). After treatment with TGD for 48 hours, the expression of hydroxyproline was performed, according to the manufacturer's instructions. Absorbance was measured at 550 nm, using an Epoch multivolume spectrophotometer system (BioTek). The results were compared with a standard curve constructed using titrating standards.

### 2.14 Enzyme‐linked immunosorbent assay

The secretion of monocyte chemoattractant protein (MCP)‐1, interleukin (IL)‐6, transforming growth factor β1 (TGF‐β1) and tumour necrosis factor (TNF)‐α in the culture supernatants of CFs with TGD treatment were determined through ELISA using commercially available kits (RD Systems, Minneapolis, MN, USA), according to the manufacturer's instructions. Absorbance was measured at 450 nm/570 nm, using an Epoch multivolume spectrophotometer system (BioTek). The results were compared with a standard curve constructed by using titrating standards.

### 2.15 Western blot

Marker (Cell Signaling Technology, Beverly, MA, USA) and 30 μg of protein from samples were separated on 10% Sodium dodecyl sulphate (SDS) gels through SDS‐PAGE. Separated protein was transferred on a polyvinylidene difluoride (PVDF) membrane that was blocked at room temperature for 1 hour in Tris‐buffered saline with 0.2% Tween 20 (TBS‐T) containing 5% skim milk and probed with primary antibodies overnight at 4°C. The diluted concentration of the primary antibodies was as follows (Abcam or Cell Signaling Technology): collagen I: 1:500; collagen III: 1:500; α‐SMA: 1:500; TGF‐β1: 1:1000; mothers against decapentaplegic homologue 3 (Smad3): 1:500; matrix metalloproteinase 9 (MMP‐9): 1:500 and tissue inhibitor of metalloproteinase 1 (TIMP‐1): 1:500; Glyceraldehyde 3‐Phosphate Dehydrogenase (GAPDH): 1:1000. The secondary antibodies (Cell Signaling Technology) included horseradish peroxidase (HRP)‐labelled and were diluted at 1:1000 and incubated for 1 hour at room temperature. Protein bands on Western blots were visualized using ECL Plus (Millipore, Billerica, MA, USA). Relative band densities of proteins on Western blots were normalized against GAPDH. The final results are expressed as fold changes by normalizing the data to the control values.

### 2.16 Data analysis and statistics

Data were expressed as the mean ± SD, except for the AF duration, which was expressed as the median and interquartile range (25%‐75%). Differences between normally distributed variables were examined through one‐way ANOVA; post hoc analyses were performed with the Bonferroni correction of multiple comparison. Differences between non‐normally distributed variables were examined using Kruskal‐Wallis ANOVA; post hoc analyses were performed with Dunn's test. All data analysis was performed with SPSS statistical software (SPSS, IL, USA). Statistical significance was defined as *P *<* *0.05.

## 3 RESULTS

### 3.1 TGD reduces AF susceptibility in rats with post‐MI

**Figure 1:**
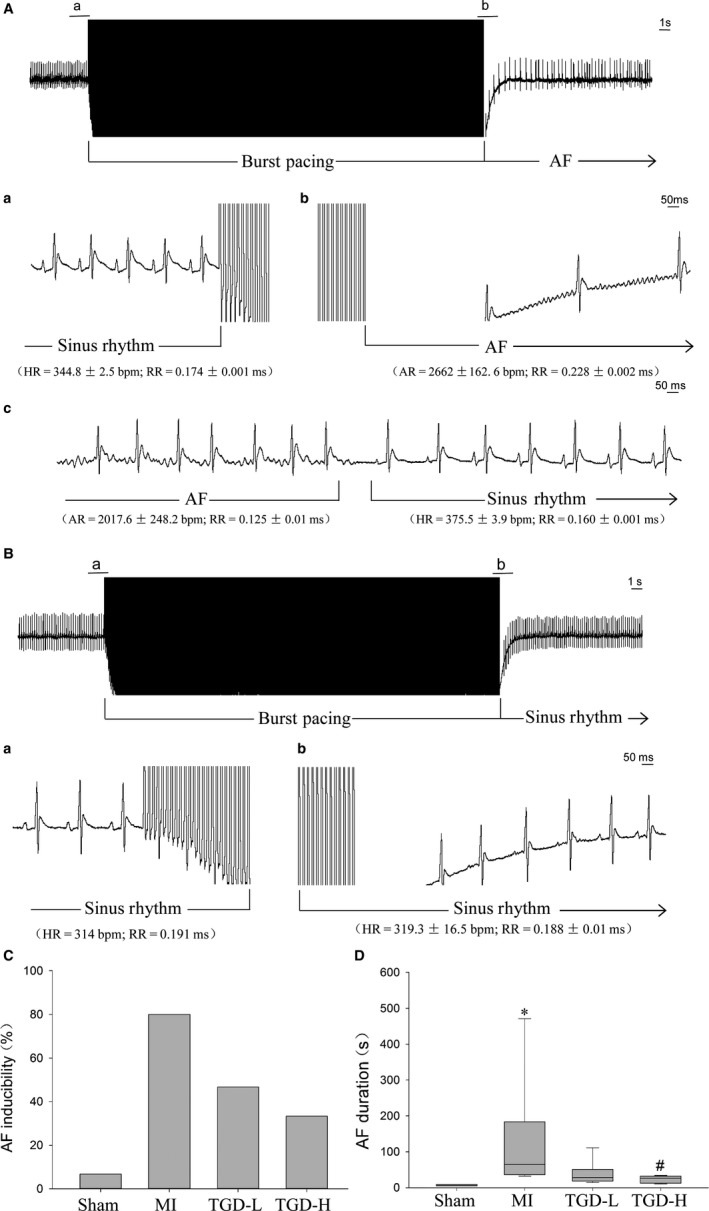
Tongguan capsule‐derived herb (TGD) inhibits atrial fibrillation (AF) inducibility and duration in post‐myocardial infarction (MI) rats. A, An example of an induced AF episode. Before the burst (a), the rat was in sinus rhythm. After termination of the burst (b), the rat displayed an irregular atrial rhythm, with an irregular ventricular response. After seconds (c), the AF episode terminated spontaneously, and the sinus rhythm resumed. B, An example of a non‐induced AF episode. After termination of the burst (b), the rat also displayed sinus rhythm. C, TGD inhibits AF inducibility in post‐MI rats (n* *=* *15 rats/group). D, TGD decreases AF duration in post‐MI rats (n* *=* *15 rats/group). HR, heart rate; RR, R‐wave to R‐wave interval; AR, atrial rate. *P < 0.05 vs sham group; ^#^ *P *< 0.05 vs MI group

Figure 1A shows a representative example of induced AF electrocardiogram. AF occurred after the termination of the burst pacing (Figure 1Ab). The AF terminated spontaneously, and sinus rhythm resumed (Figure 1Ac). However, the non‐induced AF rats also displayed sinus rhythm after the burst (Figure 1Bb). AF inducibility was higher in the rats with MI (12/15, 80%) than in the sham rats (1/15, 6.7%, Figure 1C). Treatment with TGD decreased the inducibility (7/15, 46.7% in TGD‐L vs 80% in MI; 33.3% in 5/15, TGD‐H vs 80% in MI). The mean duration of the AF episode was longer in the rats with MI than in the sham rats (*P *<* *0.05, Figure 1D). TGD decreased the AF duration in the TGD‐H group (*P *<* *0.05). The atrial effective refractory period (ERP) was shorter in the rats with MI than in the sham rats (*P *<* *0.01, Supporting Information). The ERP in TGD‐treated rats was longer than in rats with MI (TGD‐L, *P *<* *0.05; TGD‐H, *P *<* *0.01).

### 3.2 TGD improves the cardiac function and LA conduction function in rats with post‐MI

**Figure 2:**
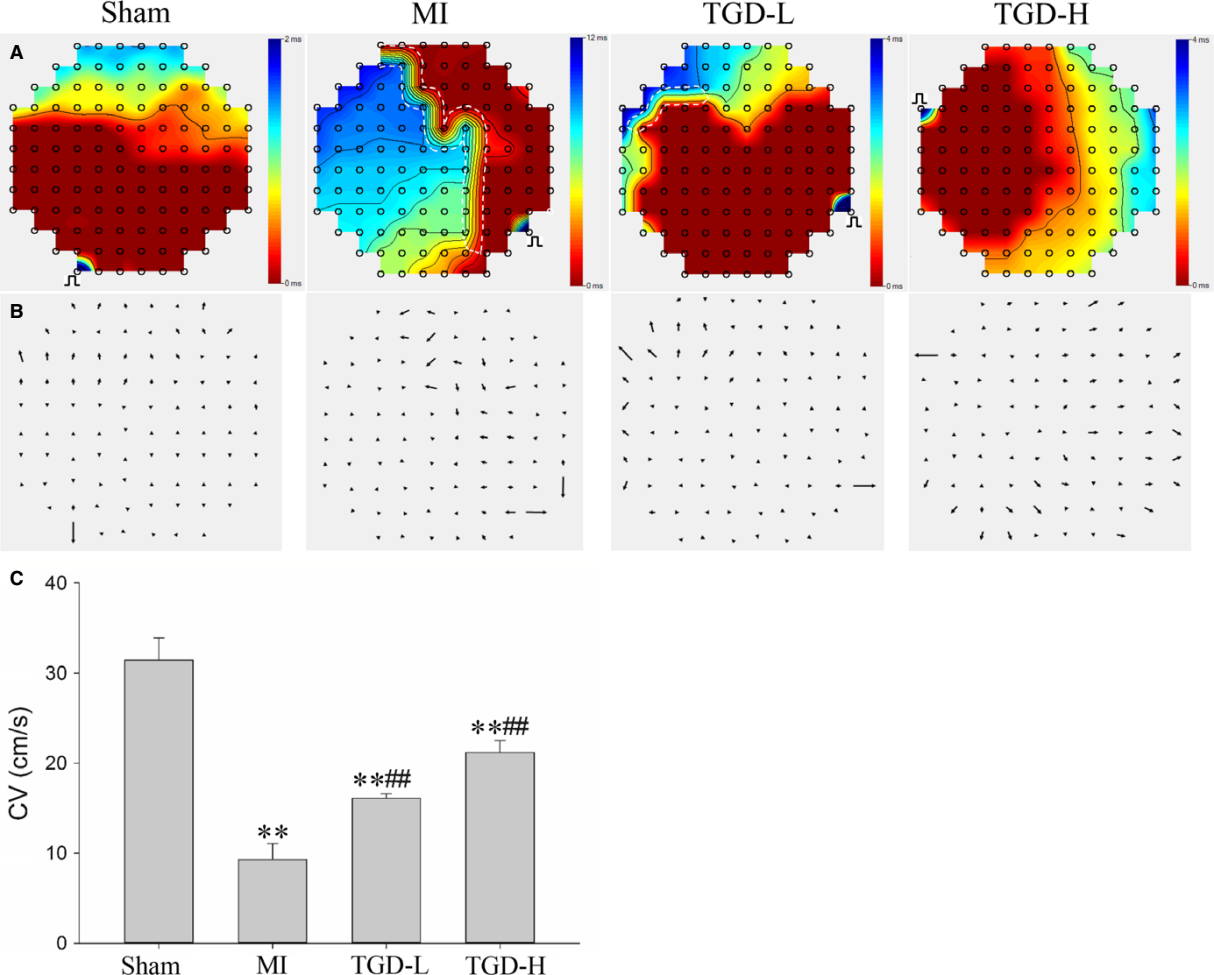
Tongguan capsule‐derived herb (TGD) improves left atrial conduction function in post‐myocardial infarction (MI) rats. A, Representative isochronous maps from multielectrode array recordings. Areas of isochronal crowding were found in the MI group. The degree of crowding decreased in the TGD‐treated rats. The black pulse symbol indicates the site of the stimulus electrode. The white dashed lines indicate the areas of conduction block. B, Representative conduction heterogeneity maps. Conduction was more heterogeneous in the rats with MI compared with the sham and TGD groups. C, Conduction velocities in the four groups. TGD improves the conduction velocity (n* *=* *5 rats/group). **P < 0.01 vs sham group; ^##^ *P *< 0.01 vs MI group

Echocardiogram showed that TGD‐treated rats significantly increased EF (*P *<* *0.01, Supporting Information) and fractional shortening (FS) (*P *<* *0.01, Supporting Information) compared with the MI group as well as declined left ventricular internal diameter at diastole and left ventricular internal diameter at systole. The isochronal maps demonstrated a typical large conduction block zone in the LA epicardium, which could block wave propagation in the MI group (Figure 2A). The activation, which was located distally, propagated to the block zone. No (or small) conduction block zone was observed in the sham and TGD‐treated groups. The MI group had more heterogeneous conduction compared with the sham and TGD‐treated groups, as demonstrated by conduction vector maps (Figure 2B). The conduction velocity was lower in the MI group than in the sham group (*P *<* *0.01, Figure 2C). This decrease was reversed after administering TGD, with a recovery of conduction velocity observed in both the TGD‐L and TGD‐H groups (*P *<* *0.01). These results indicated that TGD improved cardiac function and LA conduction function after MI.

### 3.3 TGD inhibits LA fibrosis and increases the expression of connexin 43 in rats with post‐MI

**Figure 3:**
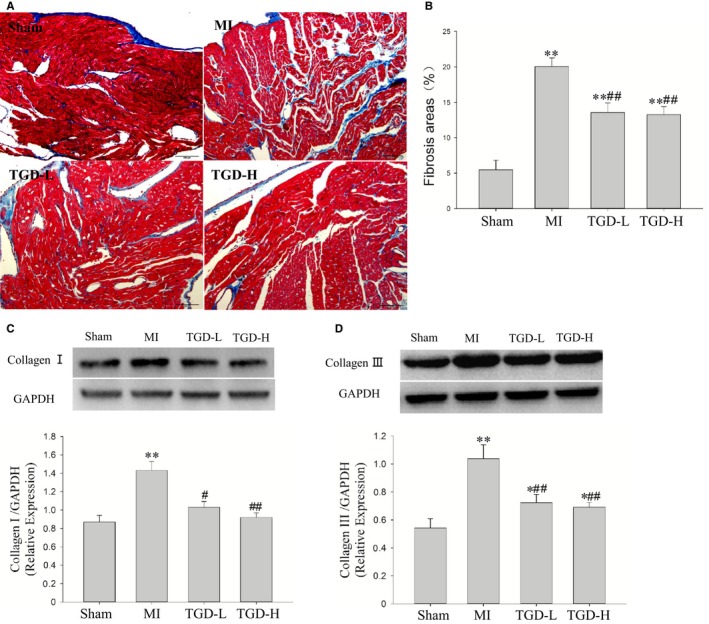
Tongguan capsule‐derived herb (TGD) inhibits left atrial fibrosis in post‐myocardial infarction (MI) rats. A, Representative images of left atrial (LA) fibrosis (Masson staining, which stains fibrosis blue and viable muscle red, scale bar: 100 μm). B, Statistical results of LA fibrosis. TGD reduces fibrosis‐positive area (n* *=* *5 rats/group). C, Western blot analysis of the protein expression of collagen I. TGD reduces the protein level of collagen I in the left atrium (n* *=* *5 rats/group). D, Western blot analysis of the protein expression of collagen III. TGD reduces the protein level of collagen III in the left atrium (n* *=* *5 rats/group). *P < 0.05, **P < 0.01 vs the sham group; ^#^ *P *< 0.05, ^##^ *P *< 0.01 vs the MI group

The Masson trichrome‐stained heart sections confirmed that MI led to increased fibrosis in the left atrium (*P *<* *0.01, Figure 3A). Figure 3B shows that TGD‐L and TGD‐H decreased the fibrotic areas (*P *<* *0.01). The deposition of cardiac collagen types I and III was evaluated through Western blot analysis to investigate atrial fibrosis. MI increased the deposition of collagen types I and III (*P *<* *0.01, Figure 3C,D). TGD treatment significantly reduced the deposition (*P *<* *0.01). The expression of connexin (Cx) 43 was significantly reduced in MI rats (*P *<* *0.01, Supporting Information). TGD treatment increased the immunohistochemical positive areas of Cx43 (TGD‐L, *P *<* *0.05; TGD‐H, *P *<* *0.01) and Cx43 protein expression (TGD‐L, *P *<* *0.05; TGD‐H, *P *<* *0.01, Supporting Information) compared with MI group.

### 3.4 TGD inhibits the expression of collagen and proliferation of cardiac fibroblasts in vitro

**Figure 4:**
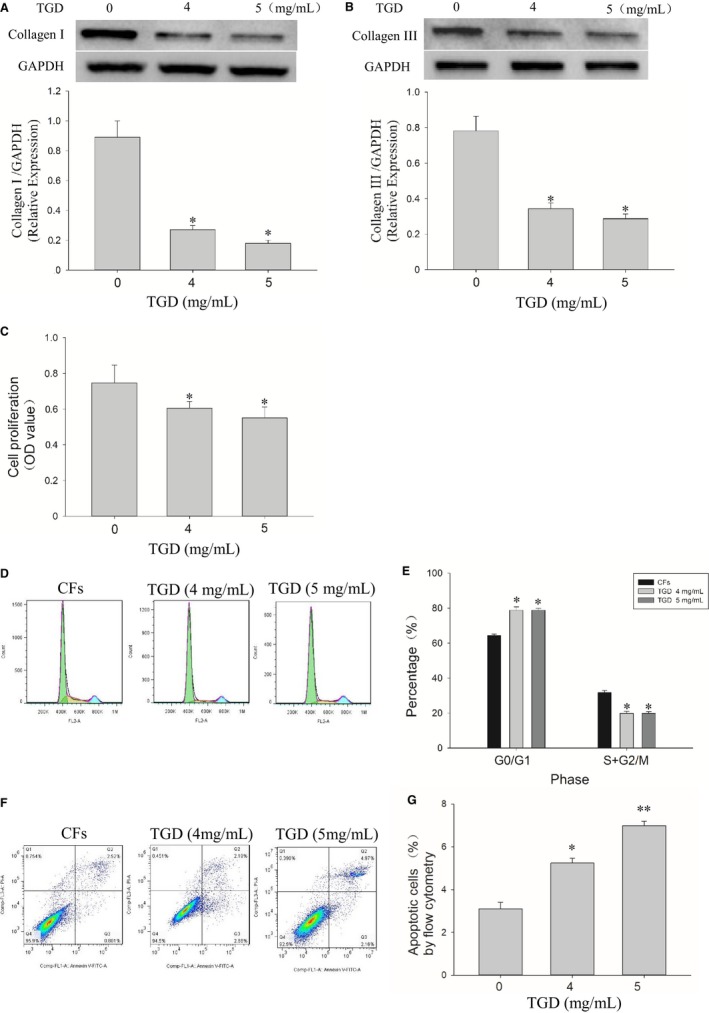
Tongguan capsule‐derived herb (TGD) inhibits cardiac fibroblasts (CFs) collagen expression and proliferation in vitro. A, Western blot analysis of the protein expression of collagen I. TGD reduces the collagen I protein expression in vitro (n* *=* *5 independent cell samples/group). B, Western blot analysis of the protein expression of collagen III. TGD reduces the collagen III protein expression in vitro (n* *=* *5 independent cell samples/group). C, The bar graph of the proliferation rate. TGD inhibits the proliferation of CFs (n* *=* *6 independent samples/group). D,E, Flow cytometry analysis of cell cycle revealed that TGD treatment induces cell cycle arrest in the G1 phase in CFs (n* *=* *4 independent samples/group). F, CFs apoptosis in different experimental groups detected using flow cytometry. CFs were labelled with Annexin V–FITC and propidium iodide. Representative images are shown. Q1, Q2, Q3, and Q4 represent necrosis, late‐stage apoptosis, early‐stage apoptosis and normal cells respectively. G, Q2 and Q3 were chosen to analyse the change in the apoptosis rate in different experimental groups (n* *=* *4 independent samples/group). *P < 0.05, **P < 0.01 vs the control group

Collagen synthesis in CFs is the most important step in the development of cardiac fibrosis. TGD significantly decreased the protein levels of collagen I and collagen III in CFs (*P *<* *0.05, Figure 4A,B). The MTT assay showed that TGD inhibited CFs proliferation (Figure 4C, *P* < 0.05). The effect of TGD on cell cycle progression was evaluated to determine the mechanism underlying TGD‐regulated CFs proliferation (Figure 4D). The result indicated that TGD treatment induced cell cycle arrest in the G0/G1 phase in CFs (*P *<* *0.05, Figure 4E). The CFs apoptosis was determined through the Annexin V–propidium iodide double staining method, using a flow cytometer (Figure 4F). Treatment with TGD increased the proportion of apoptotic cells in CFs (TGD 4 mg/mL, *P *<* *0.05; TGD 5 mg/mL, *P *<* *0.01). Together, these results indicated that TGD inhibited proliferation and induced apoptosis in CFs.

### 3.5 TGD inhibits FBS‐induced CFs migration

**Figure 5:**
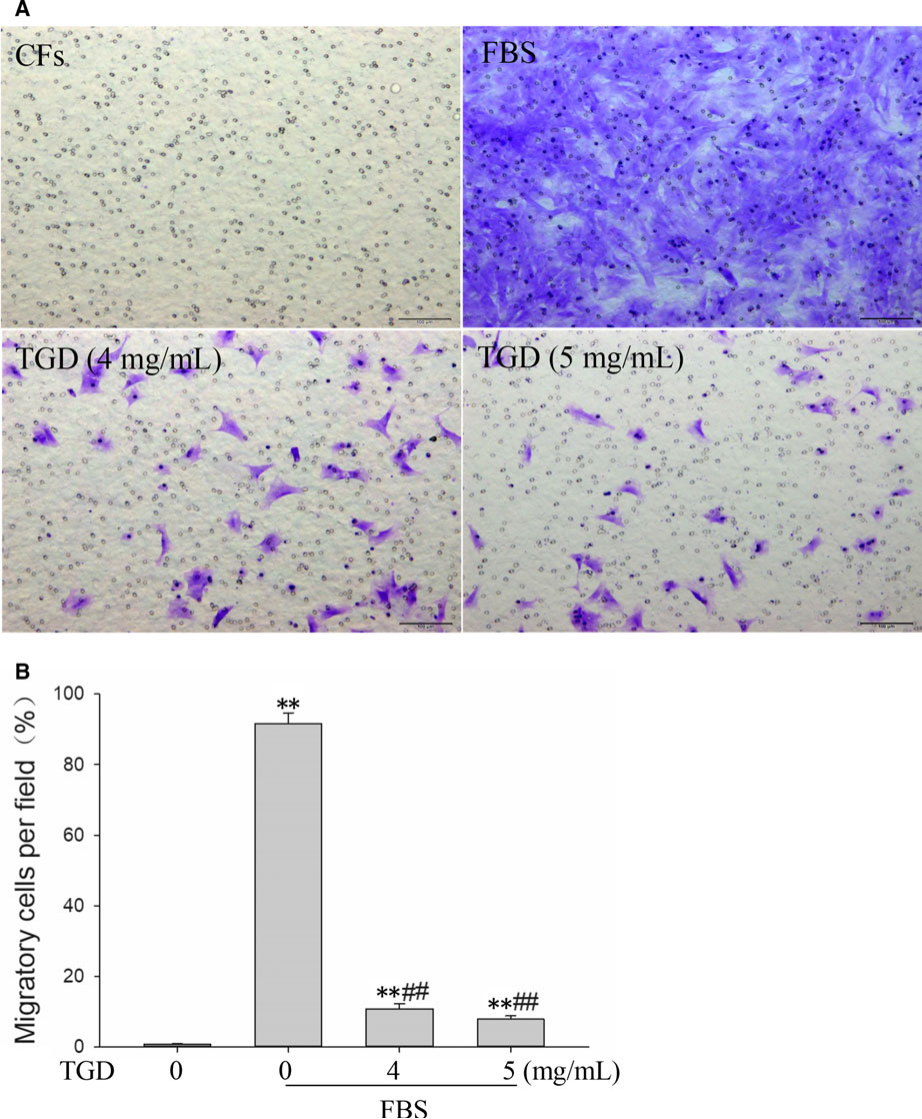
Tongguan capsule‐derived herb (TGD) inhibits foetal bovine serum (FBS)‐induced cardiac fibroblasts (CFs) migration. A, Representative images of CFs migration detected using the Transwell migration assay (scale bar: 100 μm). B, Quantitative data for CFs migration (n* *=* *4 independent samples/group). **P < 0.01 vs the control group. ^##^ *P *< 0.01 vs the FBS group

The inhibitory effects of TGD on CFs migration were examined using the Transwell assay. Representative pictures are shown in Figure 5A. FBS significantly upregulated the cell migration rate (*P *<* *0.01). Treatment with TGD significantly reversed the increase in FBS‐induced cell migration (*P *<* *0.01).

### 3.6 TGD inhibits the TGF‐β1–induced CFs differentiation into myofibroblasts

**Figure 6:**
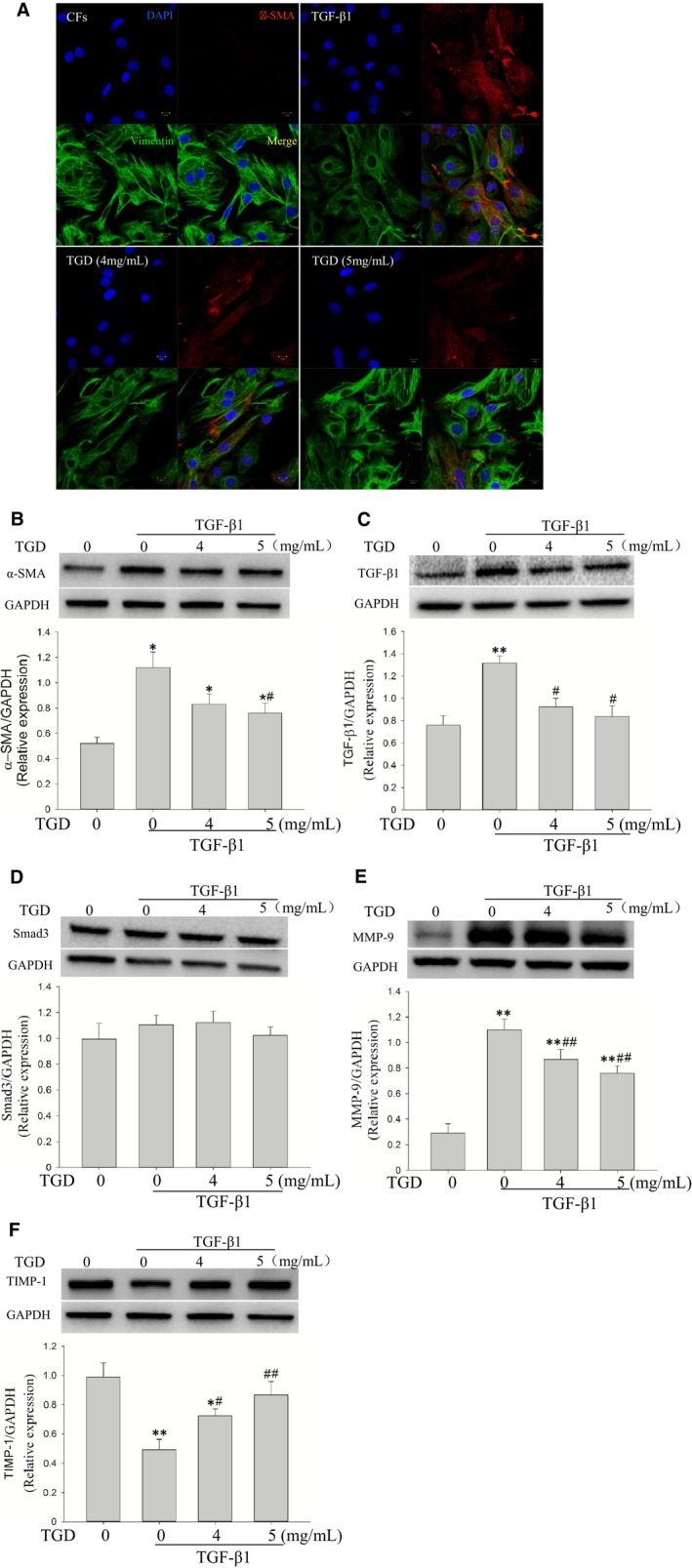
Tongguan capsule‐derived herb (TGD) inhibits TGF‐β1–induced myofibroblast transformation. A, Representative images of the expression and distribution of myofibroblast‐specific marker α‐SMA (scale bar: 30 μm). Fluorescence immunohistochemistry (using a specific α‐SMA first antibody, followed by a secondary antibody conjugated with FITC) was performed to demonstrate TGF‐β1–induced myofibroblast transformation. Nuclei were stained with DAPI. B, Western blot analysis of the protein expression of α‐SMA. TGD reduces the expression of α‐SMA protein in cardiac fibroblasts (n* *=* *5 independent samples/group). C, Western blot analysis of the protein expression of TGF‐β1. TGD reduces the expression of TGF‐β1 protein in cardiac fibroblasts (n* *=* *5 independent samples/group). D, Western blot analysis of the protein expression of Smad3. TGD has no effect on the protein level of Smad3 (n* *=* *5 independent samples/group). E, Western blot analysis of the protein expression of MMP‐9. TGD reduces the protein level of MMP‐9 (n* *=* *5 independent samples/group). F, Western blot analysis of the protein expression of TIMP‐1. TGD increases the protein level of TIMP‐1 (n* *=* *5 independent samples/group). *P <* *0.05, **P <* *0.01 vs the control group; ^#^ *P *<* *0.05, ^##^ *P *< 0.01 vs the TGF‐β1 group

Fibroblasts may respond to mechanical loading through a switch to a myofibroblastic phenotype in which they express α‐SMA.^32^ The transformation of myofibroblasts from CFs is a critical event in the initiation of myocardial fibrosis.^33^ The total cells were identified using DAPI and vimentin staining to detect the effects of TGD on TGF‐β1–induced myofibroblast transformation. The α‐SMA‐positive cells were identified through FITC staining. In the present study, the upregulation of α‐SMA was detected through immunofluorescence in TGF‐β1‐treated cells. TGD reversed the effects of TGF‐β1 (Figure 6A). Western blot analysis also showed that TGD reduced the expression of α‐SMA protein (*P *<* *0.05, Figure 6B). The TGF‐β1 pathway was vital in the transformation of myofibroblasts. Western blot analysis showed that the protein levels of TGF‐β1 were significantly greater in the TGF‐β1 group than in the control group (*P *<* *0.01, Figure 6C). TGD reduced the expression of TGF‐β1 protein compared with the TGF‐β1 group (*P *<* *0.05). Smad3 showed no differences between the four groups (*P > *0.05, Figure 6D). Figure 6E shows that the expression of MMP‐9 significantly increased in the TGF‐β1 group compared with the control group (*P > *0.05). TGD decreased the level of MMP‐9 compared with the TGF‐β1 group (TGD 4 mg/mL, *P *<* *0.05; TGD 5 mg/mL, *P *<* *0.01). Conversely, the expression of TIMP‐1 decreased in the TGF‐β1 group (*P *<* *0.05). TGD increased the level of TIMP‐1 (*P *<* *0.05, Figure 6F). The data demonstrated that TGD inhibited the myofibroblast differentiation induced by TGF‐β1.

### 3.7 TGD inhibits CFs secretion in vitro

**Figure 7:**
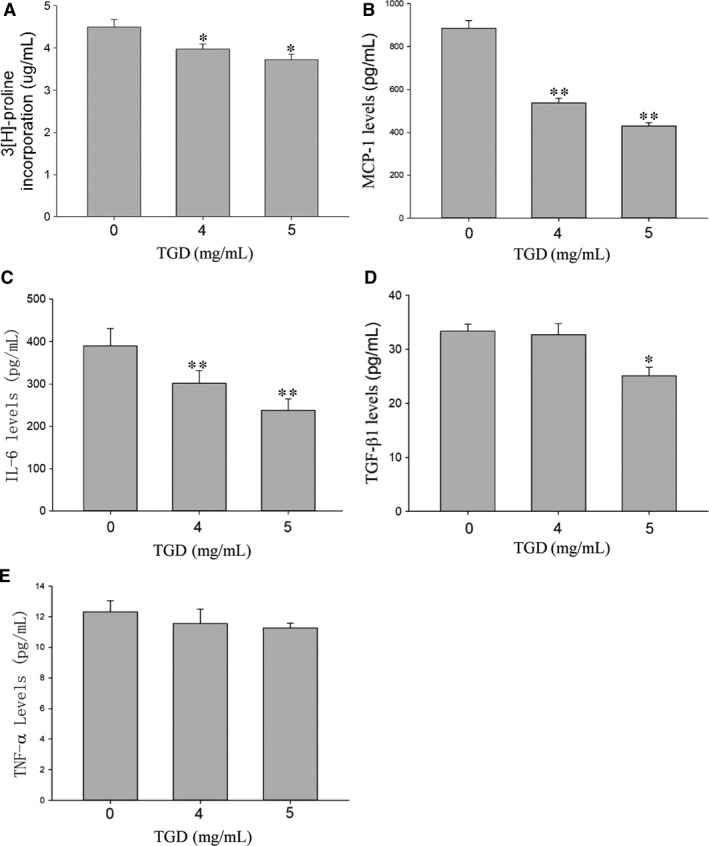
Tongguan capsule‐derived herb (TGD) inhibits cardiac fibroblasts secretion in vitro. A, Hydroxyproline assay in the cell culture supernatants. TGD inhibits collagen secretion (48 h, n* *=* *5 independent samples/group). B‐E, ELISA of MCP‐1, IL‐6, TGF‐β1 and TNF‐α in the cell culture supernatants. TGD reduces the level of MCP‐1, IL‐6 and TGF‐β1, without TNF‐α (48 h, n* *=* *5 independent samples/group). *P <* *0.05, **P <* *0.01 vs the control group

As collagen is a key component of the extracellular matrix regulated by myocardial fibroblasts, the secretion of collagen by CFs was investigated using the hydroxyproline assay. Figure 7A shows that the exposure of CFs to TGD decreased the total collagen levels (*P *<* *0.05). Several cytokines secreted by CFs had direct effects on fibrosis and conduction velocity. The secretion levels of MCP‐1, IL‐6, TGF‐β1 and TNF‐α from the cell culture supernatants were examined by ELISA. TGD decreased the levels of MCP‐1, IL‐6 and TGF‐β1 (Figure 7B‐D). The level of TNF‐α in TGD‐incubated supernatants showed no significant change compared to the control group (*P > *0.05, Figure 7E). These results indicated that TGD inhibited the cytokine secretion of CFs.

## 4 DISCUSSION

This study found that TGD, a Chinese herb medicine, could reduce AF susceptibility in post‐MI rats by inhibiting LA fibrosis via modulating CFs. The primary findings of this study were as follows: (a) TGD decreased the AF inducibility rate and shortened the duration 5 weeks after MI; (b) TGD reduced LA fibrosis and improved the atrial conduction function 5 weeks after MI; and (c) TGD reduced the proliferation, migration, differentiation of myofibroblasts, as well as cytokine secretion, and promoted CFs apoptosis, which was associated with reduced cardiac fibrosis in vitro. This novel study systematically investigated the anti‐AF effect of a Chinese herb, using fibroblasts as the target. The findings indicated that TGD reduces susceptibility to AF and improves LA conduction function in rats with post‐MI by inhibiting LA fibrosis via modulating CFs.

Atrial fibrillation is generally considered to be a complicated and progressive condition. Ectopic (triggered) activity and re‐entry are major and necessary arrhythmogenic mechanisms in AF.^34^, ^35^ Each AF episode requires initiation by triggers on a vulnerable substrate. The ion channel determinants govern AF initiation, and the structural remodelling stabilizes AF‐maintaining re‐entrant mechanisms.^36^ Development and progression of LA fibrosis are the hallmark of structural remodelling in AF and are considered to be the substrate for AF perpetuation.^37^ Fibrotic transformation of atrium results in the deterioration of atrial conduction. This increases anisotropy of impulse propagation and builds boundaries that promote re‐entry in the atrial walls, which may be directly related to the mechanisms responsible for maintaining AF.^36^, ^38^ Pharmacologic therapy targeted at the fibrotic substrate itself may be important in managing AF. Drugs that prevent the LA fibrosis, such as pirfenidone,^10^ pioglitazone^11^ and Irbesartan^12^ can effectively inhibit AF susceptibility in different animal models. In the present study, TGD significantly decreased fibrotic areas and the deposition of collagen type I/III in the left atrium, compared with rats with MI. This finding strongly proved that TGD can reduce LA fibrosis. TGD also improved the LA conduction velocity and homogeneity, decreased the AF incidence and shortened the duration, particularly in the TGD‐H group, similar to the effects of other antifibrotic drugs.^10^, ^11^ Therefore, it was concluded that TGD decreased the AF susceptibility in post‐MI rats by improving atrial conduction function via preventing fibrosis in the left atrium.

After HF, the effect of improving the ventricular remodelling or cardiac function on AF is still inconclusive.^27^ CFs, the largest cell population in the heart, are known as the main effector cells responsible for cardiac fibrosis. After injury, various peptide growth factors stimulate fibroblasts to migrate to the wound site and proliferate to reconstitute the various connective tissue components.^39^ Fibroblasts are activated and differentiated into myofibroblasts that proliferate and cause fibrosis as a component of the wound‐healing response.^39^ In most organs, active fibroblasts undergo apoptosis and disappear following the completion of tissue repair, while in the heart, myofibroblasts have been shown to persist in atrium or mature infarct scars.^27^, ^40^ Myofibroblasts are responsible for excessive deposition of the extracellular matrix. Myofibroblasts also form electrical coupling (through connexin) with cardiomyocytes in the heart.^41^ Existing myofibroblasts may actively contribute to the structural and electrical remodelling leading to the development of cardiac arrhythmias.^9^ Therefore, a novel antiarrhythmic therapeutic approach targeted at the CF population is needed.^40^, ^42^ We tested the expression of myofibroblasts using a specific marker α‐SMA in vitro. TGD decreased the expression of α‐SMA, collagen I and collagen III in CFs. These findings demonstrated that TGD inhibited the differentiation of fibroblasts into myofibroblasts in vitro.

Fibroblasts and myofibroblasts produce and secrete a number of chemical mediators, which help to maintain the inflammatory response to injury.^43^, ^44^ The effects of chemical mediators have been studied by treating cultured myocytes with fibroblast‐conditioned media.^44^ LaFramboise has found that neonatal fibroblast chemical mediators affect the size, contractile capacity and phenotype plasticity of cardiac myocytes in cultures.^45^ Paracrine factors also demonstrate direct electrophysiological effects, contributing to conduction slowing.^46^ Vasquez has reported cardiac injury significantly alters the profile of paracrine factors released by fibroblasts, potentially increasing arrhythmogenesis.^47^ The present study found that TGD decreased the level of MCP‐1, IL‐6 and TGF‐β1 in the cell culture supernatants, indicating that TGD inhibited the cytokine secretion of CFs in vitro.

Several limitations of the present study should be highlighted. First, we provided no information on the exact mechanism through which TGD reduced arrhythmia in our study. Ion channels in cardiomyocytes are as important as atrial fibrosis in AF occurrence after MI. We could not exclude the effect of TGD on ion channels in our model. TGD increases the protein expression of Cx43 in the left atrium (Supporting Information), which may be another mechanism through which TGD reduces the susceptibility of AF. Second, TGD is a complex mixture of herbs, and little is known about the effects of the individual components and their interaction with one another. As TGD is composed of diverse herbal medicine ingredients, the compound(s) in TGD that are responsible for the effects observed in this study must to be further investigated.

## 5 CONCLUSION

In recent years, cardiovascular medicine has undergone a major paradigm shift, transforming its focus from symptom‐based therapy to prevention of the elements in the cascade of pathogenesis in cardiovascular disease.^48^ Chinese herbs have been widely used in Chinese clinical cardiovascular therapy, with the notable advantages of being multi‐component and multi‐target.^49^ In conclusion, the present study showed that TGD decreases AF occurrence after MI by preventing LA fibrosis by inhibiting the proliferation, migration, differentiation and cytokine secretion of CFs. Hence, targeting the CF population may be a novel antiarrhythmic therapeutic approach, and Chinese medicine, particularly TGD, could play a beneficial role.

## CONFLICT OF INTEREST

The authors declare no conflicts of interest.
