# Supplementary material for: Tongguan capsule‐derived herb reduces susceptibility to atrial fibrillation by inhibiting left atrial fibrosis via modulating cardiac fibroblasts
Source: J Cell Mol Med. 2018 Nov 19;23(2):1197–210. doi: 10.1111/jcmm.14022 (PMC6349173; doi:10.1111/jcmm.14022)
Supplement: Supplementary file 1 [file JCMM-23-1197-s001.doc]

**Tongguan capsule-derived herb reduces** **susceptibility to atrial fibrillation by inhibiting left atrial fibrosis via modulating cardiac fibroblasts**

Shiyu Ma^a, b, 1^, Jin Ma^c, 1^, Liheng Guo^a,b^, Junqi Bai^d^, Shuai Mao^a,b^, Minzhou Zhang^a,b,*^

**Supplementary file**

**Quality control of Tongguan Capsule–derived herb (TGD) through high-performance liquid chromatography (HPLC)**

Calycosin-7-O-β-D-glucoside, Formononetin-7-O-β-D-glucoside, Calycosin, Salvianolic acid B are the main constituents of the herbs in the TGD formula. Regarding to the identification methods of the main constituents of the four Chinese herbs in the Chinese Pharmacopoeia, we used the four constituents analysis method for quantification of the TGD through HPLC. The test solution (TGD powder: 688 mg, extracted from 10 g prescription herbs) was dissolved in 100 ml ultra-pure water (Aqua Millipore) and filtered (Millex-LCR, Hydrophilic PTFE, 0.22 μm VWR International, LLC.). The reference standard solution (1 mL) contains a mixture of Calycosin-7-glucoside (CAS No.20633-67-4) 0.20 mg, Formononetin-7-O-β- D-glucoside (CAS No.486-62-4) 0.2 mg, Calycosin (CAS No. 20575-57-9) 0.1 mg, and salvianolic acid B (CAS No.115939-25-8) 1.8 mg dissolved in methanol and filtered. The test and reference standard solution were directly subjected to analytical HPLC on ACQUITY TM High Performance LC (λ = 254nm) in-line degasser, Agilent 1260 LC system (Agilent company, USA) on an RP-18 stationary phase Luna C18 (2), 5 µm, 4.6 ×250 mm (Phenomenex company, USA). Mobile phase: binary gradient of water containing acetonitrile (A) and 0.1% phosphoric acid (B): t 0~22 min 12% A→20% A, t 22~35 min, 20% A→30% A. Flow rate: 1.8 ml/min. Injection volume: 5 µl. Column temperature: 30 °C.

**Chromatography and mass spectrometry conditions**

Ultra high-performance liquid chromatography (UHPLC) analyses were performed on an ACQUITY^TM^ UHPLC I-Class system equipped with a binary solvent system and an auto-sampler (waters, Milford, USA). Chromatographic separation was performed on a Phenomenex Kinetex C 18 column (2.1mm × 100 mm, 1.7 µm) at room temperature. The mobile phase consisted of acetonitrile (A) and water containing 0.1% formic acid (B) and the elution gradient was set as follows: 5% A (0 min), 17% A (2 min), 19% A (16 min), 27% A (22 min), 37% A (45 min), 65% A (60 min). The mobile phase flow rate was 400 µL/min. An aliquot of 2µL was injected into the UHPLC system.

Mass spectrometry (MS) data were acquired on an AB SCIEX Triple TOF 5600 (AB Sciex Pte. Ltd., Singapore). The system was controlled with AB SCIEX Analyst TF (Version 1.7) software (AB Sciex Pte. Ltd., Singapore, Singapore). The MS conditions were as follows: electrospray ionization, the ion spray voltage was set at +/- 4500 V. The capillary voltage was fixed at 15 V, and the temperature was maintained at 500 ^o^C. Declustering potential, +/-80 V. Nitrogen was used as the nebulizer and the auxiliary gas, and the nebulizer (gas 1), the heater gas (gas 2) and the curtain gas were set to 50, 50, 40 psi, respectively. The collision energy (CE) was set at +/-45 eV and the CE spread was 15 eV, enabling us to obtain an average enhanced product ion scan spectrum when the CE was +/-30, +/-45, and +/-60 eV. For the full MS-information dependent acquisition-8MS/MS analysis, the scan range was operated with the mass m/z 120 to m/z 1200, and with a 100-ms accumulation time. Furthermore, an automated calibration delivery system was used to regulate the MS and the MS/MS automatically. Characteristic UHPLC-MS peaks (profile) are presented in Figure S2.

**Echocardiogram**

One week or five weeks after surgery, all rats were anaesthetized with 2% sevoflurane. Transthoracic echocardiography was performed using a 21-MHz phased-array probe (Vevo 2100, VisualSonics Inc, Canada). The echocardiography was recorded in the parasternal short axis B-mode and M-mode views at the level of the papillary muscles to obtain ejection fraction (EF), and in the parasternal long axis view to measure left ventricular end-systolic and end-diastolic internal diameters (LVIDs and LVIDd). Each echocardiographic variable was determined in at least four separate left ventricular images taken from the same heart.

**Electrophysiological investigation**

Regular pacing and standard S1S2 programmed pacing protocols were used to determine the atrial effective refractory period (ERP). The intensity of the current pulse was twice that of the threshold. ERP was measured by introducing S2 extra stimulus, with 4-ms decrements following eight regulatory S1-S1 stimuli of 120 ms, and it was defined as the longest S1-S2 interval at which S2 failed to induce a propagated response. We measured ERP two times on each animal, and the average of them was defined as ERP of each animal.

**Immunohistochemical assay**

The heart sections were stained with Cx43 (1:200; Cell Signaling Technology) and rabbit immunoglobulin G or rabbit serum instead of primary antibody was used as a negative control. The peroxidase activity was visualized using diaminobenzidine, and the sections were counterstained with haematoxylin, and 20× magnification images were obtained using a DP 72 camera under a BX53 microscope (Olympus, Tokyo, Japan). Five fields were randomly chosen from each slide. We quantified the percentages of positive staining area using CellSens Dimension 1.16 software (Olympus, Tokyo, Japan).

**Results**

**Quality control of the TGD by HPLC**

The HPLC chromatogram of the mixed reference solution and the test sample (TGD) solution are shown in Figure S1. The test solutions of TGD were repeatedly measured (i.e., six times) using HPLC methods, and the results of relative standard deviation (RSD) were as follows: Calycosin-7-O-β-D-glucoside, 0.59%; Formononetin-7-O-β- D-glucoside, 0.77%; Calycosin, 1.08%; and Salvianolic acid B, 0.88%. The results show that the test method has good repeatability. Three sample batches of TGD were accurately tested and measured the quantity of four constituents using the same methods. The results of RSD were as follows: Calycosin-7-O-β-D-glucoside, 5.16%; Formononetin-7-O- β-D-glucoside, 4.56%; Calycosin, 3.77%; and Salvianolic acid B, 3.74% (Table S1). The results showed that the primary constituents of different batches remains stable.

**Compounds identification of TGD**

As shown in Figure S2, peaks in the UHPLC-MS fingerprints were identified and confirmed using the available reference compounds. A list of these identified peaks is provided in Table S2. The peak flow showed the index components, such as Calycosin-7-O-β-D-glucoside, rosmarinic acid, salvianolic acid B and salvianolic acid A.

**Cardiac function at one week and five weeks after surgery**

The mean EF and fractional shortening (FS) of the MI rats were markedly decreased one week after surgery. LVIDd and LVIDs of the MI rats were increased compared with the sham rats. There was no significant different in cardiac function in the MI rats (Table S3). With the progression of MI, all cardiac function parameters in the MI group were further worsened five weeks after surgery. However, the rats that received TGD-treatment for four weeks had significantly increased EF and FS compared with the MI group, as well as declined LVIDd and LVIDs (Table S3).

**The ERP in four groups five weeks after surgery**

The ERP was shorter in the rats with MI than in the sham rats (*P* < 0.01, Table S4). The ERP in the TGD-treated rats were longer than in the rats with MI (TGD-L, *P* < 0.05; TGD-H, *P* < 0.01)

**TGD increased the expression of Cx43 in the left atrium**

The Cx43-positive area in the left atrium was significantly reduced in the MI group (Figure S3A). TGD treatment increased the positive areas of Cx43 (Figure S3B). The Western blot analysis showed a clear decrease in the protein expression of Cx43 in the MI group compared to in the sham group. TGD increased the protein expression of Cx43 in the left atrium (Figure S3C).

**
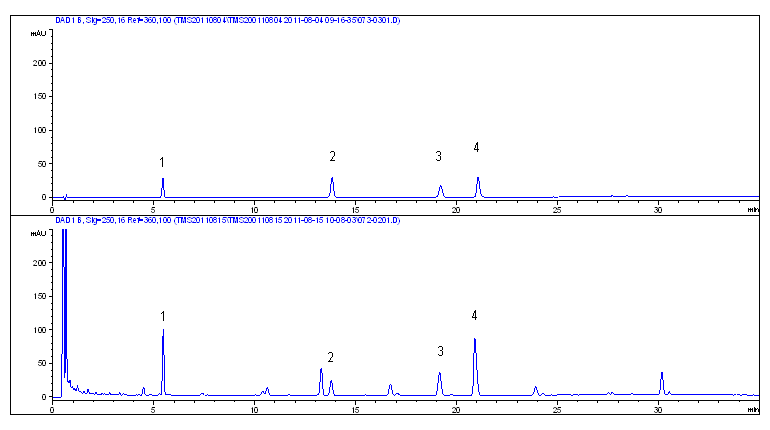
**

A

B

**FIGURE S1** Quantification of the TGD by HPLC. A shows the Representative HPLC chromatogram of the mixed reference solution. B shows the Representative HPLC chromatogram of the test sample (TGD) solution. (1) Calycosin-7-O-β-D-glucoside, (2) Formononetin-7-O-β-D-glucoside, (3) Calycosin, (4) Salvianolic acid B.

**TABLE S1** The quantity of four constituents in three batches sample of TGD

| **Constituents** | **20161109（mg/g）** | **20161203（mg/g）** | **20161204（mg/g）** | [**mean content**](http://www.baidu.com/link?url=gI7NSMb77rSWBFbD_wg1u_SS4VRNRQtcm_aLICmYjXqZn1gV_dkqL_i9EP3y0PqOi8qYGKEL4nil6S2ZESmY0muyNKomv57IPybvrIW-Pxh4_ipnSjFMceHvCVI_-EBE)**（mg/g）** | **RSD%** |
| --- | --- | --- | --- | --- | --- |
| Calycosin-7-glucoside | 0.0995 | 0.1103 | 0.1042 | 0.1050 | 5.16% |
| Formononetin-7-O-β-D-glucoside | 0.0272 | 0.0298 | 0.0285 | 0.0285 | 4.56% |
| Calycosin | 0.0375 | 0.0366 | 0.0348 | 0.0363 | 3.77% |
| Salvianolic acid B | 0.7898 | 0.8081 | 0.7507 | 0.7830 | 3.74% |

**
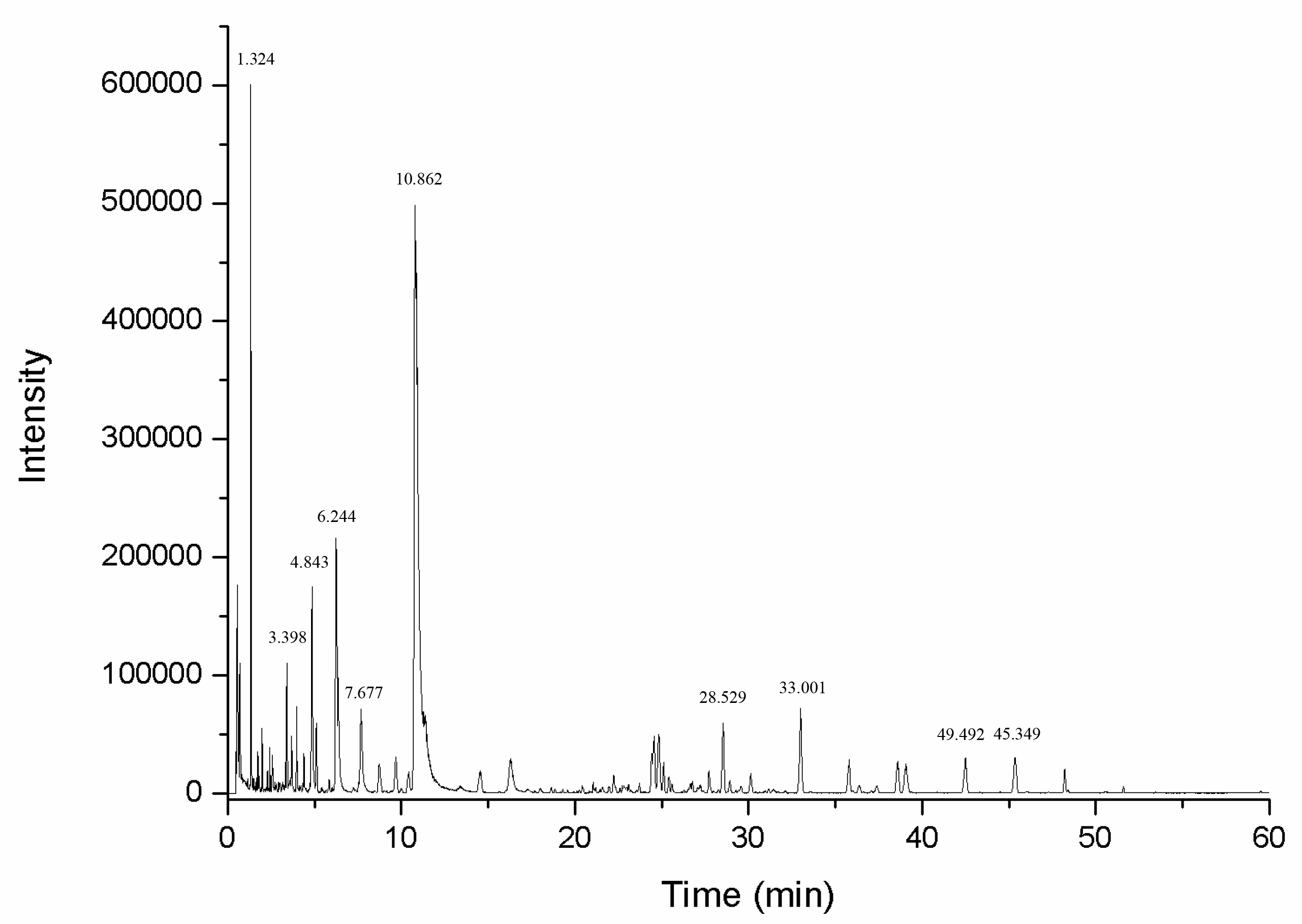
**

**FIGURE S2** The chromatograms of the isolated compounds of TGD by UHPLC-MS.

**TABLE S2** Main constituents in TGD by UHPLC-MS identification

| **o** | ***R*_t_** | **Molecular ion peak** | **Molecular formula** | **Fragmentation ion** | **Identified Compound** |
| --- | --- | --- | --- | --- | --- |
| 1 | 1.32 | 197.0460 [M-H]^-^ | C_9_H_9_O_5_ | 179.0341, 135.0444 | Dihydroxyphenyl-hydroxypropanoic acid |
| 2 | 1.73 | 205.0697 [M-H]^-^ | C_8_H_13_O_6_ | 143.0695, 115.0750 | Dihydroxyoctanedioic acid |
| 3 | 2.42 | 179.0333 [M-H]^-^ | C_9_H_7_O_4_ | 135.0434 | Caffeic acid |
| 4 | 3.39 | 491.1201 [M+HCOO]^-^ | C_23_H_23_O_12_ | 283.0598 | Calycosin-7-O-glucoside |
| 5 | 3.68 | 537.1044[M-H]^-^ | C_27_H_21_O_12_ | 295.0565 | Lithospermic acid |
| 6 | 5.10 | 187.0983 [M-H]^-^ | C_9_H_15_O_4_ | 125.0957 | 3,5-dihydroxynon-6-enoic acid |
| 7 | 6.24 | 359.0777 [M-H]^-^ | C_18_H_15_O_8_ | 161.0225, 197.0432, 179.0328, 223.0220 | Rosmarinic Acid |
| 8 | 6.35 | 717.1500[M-H]^-^ | C_36_H_29_O_16_ | 519.0866, 321.0363 | Salvianolic acid B |
| 9 | 7.67 | 537.1046 [M+HCOO]^-^  493.1148 [M-H]^-^ | C_26_H_21_O_10_ | 493.1074, 459.1154 | Salvianolic acid A |
| 10 | 7.76 | 475.1258[M-H]^-^ | C_22_H_22_O_9_ | 267.0651, 252.0414 | Formononetin-7-O-β-D-glucoside |
| 11 | 10.01 | 283.0583 [M-H]^-^ | C_16_H_11_O_5_ | 268.0343 | Calycosin |
| 10a毛蕊异黄酮苷及毛蕊异黄酮作为旱厕肝0000000000000000000000000000000000000000000000000000000000000000000000000000000000000000000000000000000000002 | 10.86 | 717.1511 [M-H]^-^ | C_36_H_29_O_16_ | 519.0866, 321.0363 | Salvianolic acid B |
| 13 | 14.58 | 845.4979[M+HCOO]^-^ 799.4903[M-H]^-^ | C_29_H_25_O_12_ | 637.4344, 475.3786 | Ethyl lithospermate |
| 14 | 28.53 | 829.4642 [M+HCOO]^-^ | C_42_H_69_O_16_ | 783.4449 | Astragaloside IV |
| 15 | 28.91 | 829.4656 [M+HCOO]^-^ | C_42_H_69_O_16_ | 783.4449 | Astragaloside IV |
| 16 | 29.58 | 931.4443 [M+HCOO]^-^ | C_41_H_68_O_14_ | 885.4541, 753.4092 | Astragaloside III |
| 17 | 33.00 | 871.4762 [M+HCOO]^-^ | C_43_H_70_O_15_ | 825.4687 | Astragaloside II |
| 18 | 35.76 | 871.4762 [M+HCOO]^-^ | C_43_H_70_O_15_ | 825.4687 | Astragaloside II |
| 19 | 48.52 | 899.4731 [M+HCOO]^-^ | C_44_H_70_O_16_ | 853.4655,721.4216, 621.0192, 377.0144 | Ophiopogonin D |

**TABLE S3** The cardiac function of four groups

|  | **Time** | **LVIDd (mm)** | **LVIDs (mm)** | **EF (%)** | **FS (%)** |
| --- | --- | --- | --- | --- | --- |
| **Sham** | 1 w | 7.40±0. 60 | 4.93±0.53 | 60.3±4.9 | 33.4±3.6 |
|  | 5 w | 8.05±0.85 | 5.32±0.48 | 60.6±6.3 | 33.8±4.7 |
| **MI** | 1 w | 8.72±0.59^▲^ | 7.13±0.67^▲▲^ | 36.0±3.8^▲▲^ | 17.8±1.8^▲▲^ |
|  | 5 w | 9.57±0.71^▲▲^ | 8.11±0.81^▲▲^ | 31.1±4.7^▲▲^ | 15.4±2.5^▲▲^ |
| **TGD-L** | 1 w | 8.78±0.64^▲^ | 7.16±0.61^▲▲^ | 36.6±5.4^▲▲^ | 18.2±1.6^▲▲^ |
|  | 5 w | 8.75±0.60^▲#^ | 6.93±0.69^▲▲##^ | 40.6±3.8^▲▲##^ | 20.8±3.1^▲▲##^ |
| **TGD-H** | 1 w | 8.85±0.74^▲^ | 7.09±0.68^▲▲^ | 35.7±4.1^▲▲^ | 18.1±2.6^▲▲^ |
|  | 5 w | 8.69±0.91^▲##^ | 6.82±0.67^▲▲##^ | 42.0±4.4^▲▲##^ | 21.4±2.4^▲▲##^ |

^▲^*P*＜0.05, ^▲▲^ *P*＜0.01 versus sham group; ^#^ *P*＜0.05, ^##^ *P*＜0.01 versus MI group.

**TABLE S4** The ERP in four groups at five weeks after surgery

|  | **Sham** | **MI** | **TGD-L** | **TGD-H** |
| --- | --- | --- | --- | --- |
| **ERP [ms] (S1S1 120 ms)** | 67.2±3.3 | 38.4±4.5^▲▲^ | 50.4±4.5^▲▲#^ | 56.8±3.3^▲##^ |

^▲^*P*＜0.05, ^▲▲^ *P*＜0.01 versus sham group; ^#^ *P*＜0.05, ^##^ *P*＜0.01 versus MI group.


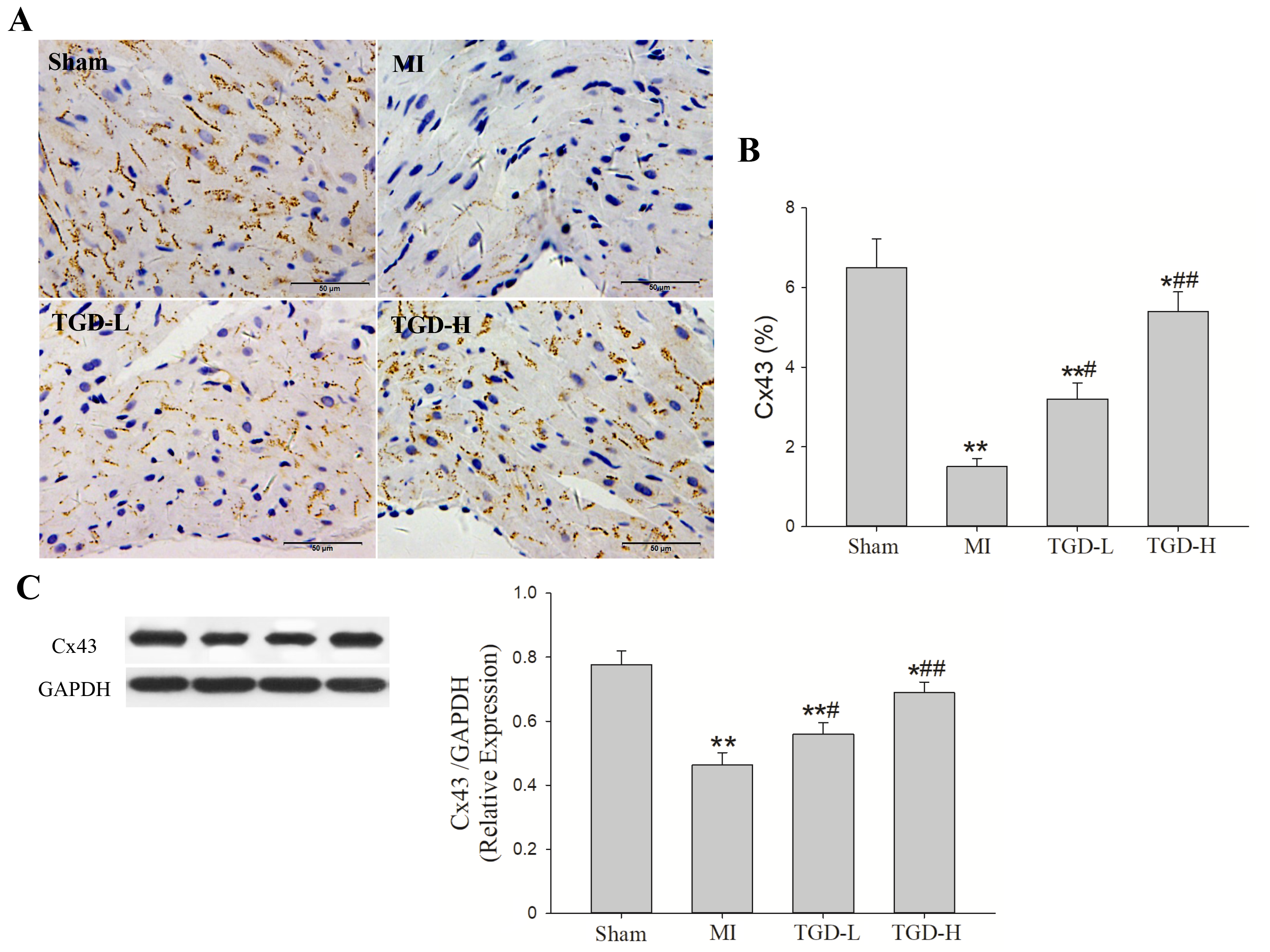


**FIGURE S3** TGD increased the expression of Cx43 in the left atrium after MI. (A) Representative figure of immunohistochemical staining of Cx43 (scale bar: 50 µm). (B) Percentage of areas of Cx43 among the three groups (*n*=5). (C) Western blot analysis of the protein expression of Cx43. TGD increased the protein level of Cx43 (*n* = 5 independent samples/group). **P* < 0.05, ***P* < 0.01 versus sham rats; ^#^*P* < 0.05, ^##^*P* < 0.01 versus MI rats.
